# A New Method of Treating Uterine Hæmorrhages after Delivery

**Published:** 1781-01

**Authors:** 


### SECTION II.

### ESSAYS AND OBSERVATIONS.

I. *A new Method of treating Uterine Hæmorrhages after Delivery. By* M. Saxtorph, M. D. *(from the* Acta Havnienſia, Vol. II.)

THIS new method, which conſiſts in the uſe of vinegar and water injected into the uterus, has ſucceeded in three caſes of menorrhagia, that had reſiſted every other means of cure. In the firft cafe the flow of blood from the uterus had continued 4 hours, and cold drink, as well as cold applications to the abdomen had been tried without effect. No pulfe or refpiration were perceptible, and the patient was perfectly cold. In this ftate a mixture of vinegar and cold water was injected into the uterus. The hæmorrhage was immediately ftopt, and the patient's pulfe and breathing returned. The reafon why injections of this fort have fo often failed in cafes of menorrhagia and fluor albus, is perhaps owing to their having been thrown up into the vagina only, and not into the uterus. In this patient the injection was repeated three or four times, and fhe was perfectly recovered in fourteen days. The lochia flowed in fmall quantity, and fhe fecreted but little milk.

In the fecond cafe the patient from the continuance of the uterine difcharge was troubled with convulfions and frequent fyncope, but as foon as the injection was thrown into the uterus, the menorrhagy ftopt, and fhe recovered in a fhort time.

In the third cafe the uterine hæmorrhage had been thrice ftopt by preffure and cold applications to the abdomen, but conftantly returned again, again, till at length the ſame injection was uſed as in the other caſes, and proved equally effectual.

With regard to uterine hæmorrhages it may be obſerved, that if the placenta is attached to the anterior ſurface of the uterus, its adheſion is always uncommonly ſtrong. The more that part of the uterus to which the placenta is fixed is extended during pregnancy, and the more that ſame part is in conſequence of ſuch extenſion contracted after delivery, ſo much the more eaſily, in general, will the placenta be ſeparated, and ſo much the leſs reaſon will there be to fear menorrhagia. The fundus uteri is, during pregnancy, the moſt extended, and after delivery the moſt and the quickeſt contracted; of courſe the placenta, when attached to the fundus, is always eaſily ſeparated, and it very ſeldom happens that its removal occaſions a violent flooding; on the contrary, the anterior ſurface of the uterus not being much ſtretched, and contracting little and ſlowly after delivery, the placenta when fixed to this part will, in general, adhere very ſtrongly, and its ſeparation frequently give riſe to conſiderable hæmorrhage. It will therefore be prudent for the accoucheur, as ſoon as he he perceives that the placenta adheres to the anterior ſurface of the uterus, to provide himſelf with a ſyringe, and cold water, and to be careful not to ſeparate the cake till he is convinced that the uterus begins to contract itſelf, and even then to proceed ſlowly.

